# Edith Cavell's Home-Coming

**Published:** 1919-05-31

**Authors:** 


### EDITH CAVELL'S HOME-COMING.

### With Many Illustrations.

The Monograph of the meaning and teachings of Edith Cavell's life and character, her home-coming and burial, in "The Hospital" of May 24th, 1919, has already attracted many readers. Some practitioners and every nurse may desire to have a copy for permanent preservation, and we have decided to reprint the Monograph should the demand for copies continue. They can be obtained, price 6*d.* each, by immediate personal application at "The Hospital" Office, 28-9 Southampton Street, Strand, London, W.C. 2, or by letter addressed to The Manager at that address enclosing 8*d.* in stamps, which includes postage. It is printed on art paper, the illustrations are beautifully executed and of permanent interest to all classes throughout the Empire, especially to matrons, sisters and nurses.

### SELECTED PRESS NOTICES.

The following Press notices have appeared:—

AN APPRECIATION OF HER LIFE.

"The current number of THE HOSPITAL (2d.) contains a special supplement of the Life of Edith Cavell, her home-coming, and burial. The appreciation of her life has been carefully written, and her final home-coming is illustrated with pictures of the moving scenes of the funeral procession."—*The Times.*

AN ATTRACTIVE AND BRIEF ACCOUNT OF EDITH CAVELL'S LIFE.

"In a Special Supplement to the current number of THE HOSPITAL is contained, in an attractive form, a brief account of the life of Edith Cavell, together with an admirable summary of the public testimony to the veneration in which her memory is held, which was made manifest on the occasion of the home-coming of the martyred body a few days ago. This final incident in a story of the highest significance is illustrated by a series of excellently reproduced photographs of the scenes at Dover, London and Norwich, and to study the solemn bearing of the crowds depicted therein gives insight into the profound appeal which her heroic spirit has made to the hearts of her fellow-countrymen. Miss Cavell acted as a correspondent of *The Nursing Mirror*, and in her last contribution to that journal, dated March 29, 1915, she voices that deep sympathy with suffering and admiration for courage which characterised her whole life."—*Daily Telegraph.*

EDITH CAVELL'S HOME-COMING, WITH MANY ILLUSTRATIONS.

"A study of Edith Cavell's life history and its lessons, her home-coming and burial, with many illustrations, make a noteworthy supplement to this week's issue of THE HOSPITAL."—*Daily Chronicle.*

THIS STORY SHOULD BE READ BY ALL.

"The current issue of THE HOSPITAL is unusually interesting, as it contains a Special Supplement giving a full study of Edith Cavell's life history and its lessons, her home-coming, and burial. The story is illustrated by a number of photographs, and it should be read by all who admire the heroic character of the Englishwoman whose work in Belgium will never be forgotten."—*The Morning Advertiser.*

A FULL STUDY OF EDITH CAVELL'S LIFE HISTORY AND ITS LESSONS.

"THE HOSPITAL of May 24 includes a special supplement containing a full study of Edith Cavell's life history and its lessons, with many illustrations."—*Westminster Gazette.*

